# WHO's Response to Global Public Health Threats: XDR-TB

**DOI:** 10.1371/journal.pmed.0040246

**Published:** 2007-07-31

**Authors:** Kumanan Wilson, Michael Gardam, Christopher McDougall, Amir Attaran, Ross Upshur

(This letter appears in response to the May 2007 *PLoS Medicine* Editorial: “How Is WHO Responding to Global Public Health Threats?” [1])

The travels of a patient with extensively drug-resistant tuberculosis (XDR-TB) from North America to Europe and back have heightened concerns about this growing global disease problem and the possibility of its international transmission through travel. The seriousness of this possibility raises the question of whether XDR-TB cases or outbreaks may constitute public health emergencies of international concern (PHEICs) under the 2005 International Health Regulations (IHR 2005), which became binding international law on June 15, 2007 [2]. XDR-TB may be the first case of a public health problem that tests how States Parties and the World Health Organization (WHO) apply the IHR 2005.

We believe that cases or outbreaks of XDR-TB would meet the notification criteria of the IHR 2005 as events that may constitute a PHEIC because they trigger affirmative answers to at least two of the four questions in the IHR 2005's decision instrument (Annex 2, [3]). Thus, as of June 15, 2007, States Parties must report such cases or outbreaks to WHO pursuant to the IHR 2005.

XDR-TB triggers an affirmative answer to the decision instrument's question: “Is the public health impact of the event serious?”, both because of the possibility of the event having a high public health impact and, in many instances, the need for external assistance in managing cases. XDR-TB cases would likely produce an affirmative answer to the question: “Is the event unusual or unexpected?”, because of the higher than expected case-fatality rates, particularly where XDR-TB occurs with high prevalence of HIV infection. The disease also may generate a positive answer to the question: “Is there a significant risk of international travel or trade restrictions?”, primarily because of the possibility of international media attention.

At present, XDR-TB would arguably not produce a clearly affirmative answer to the question: “Is there a significant risk of international spread?”, because epidemiological data linking XDR-TB outbreaks and cases with the international movement of people are weak. Experience with TB, multi-drug-resistant TB (MDR-TB), and XDR-TB demonstrates, however, that cross-border spread is a very real threat. XDR-TB has already spread from one province to several provinces in South Africa, and has in all likelihood crossed African borders, but confirmation is delayed because of inadequate surveillance capabilities.

It remains less certain whether the WHO Director-General would determine that XDR-TB cases or outbreaks notified to WHO would constitute a PHEIC. This would lead to the issuance of temporary recommendations to States Parties, which could include travel restrictions and use of compulsory measures such as quarantine or isolation. In February 2007, the WHO Global Task Force on XDR-TB argued against such a position because “the new regulations are aimed particularly to situations where there is a significant risk of international spread” and that temporary recommendations “are really intended for outbreaks of acute disease, rather than the ‘acute-on-chronic’ situation of MDR-TB and XDR-TB” [4].

The Global Task Force stated that, if international spread of XDR-TB were demonstrated, then standing recommendations should be considered. To some extent, the recommendations and alerts WHO has already issued on XDR-TB suggest that treating XDR-TB as a routine public health risk is not adequate. In addition, the IHR 2005 does not limit its scope of application to “acute disease” [5]. TB has never been an acute risk in the same manner as a pandemic strain of influenza, but history attests to TB's persistence as a lethal global threat, especially for vulnerable populations.

As the global XDR-TB problem becomes more severe, more robust and drastic public health actions may be required nationally and globally, including a declaration from the WHO Director-General of the existence of a PHEIC. A window of opportunity may exist now for effective international action to control the spread of the disease, and the IHR 2005 may provide a mechanism to help achieve this objective. A narrow, limited reading of the IHR 2005's scope of application could undermine its ability to contribute to this global health crisis.
